# The Use of Spinning-Disk Confocal Microscopy for the Intravital Analysis of Platelet Dynamics in Response to Systemic and Local Inflammation

**DOI:** 10.1371/journal.pone.0025109

**Published:** 2011-09-19

**Authors:** Craig N. Jenne, Connie H. Y. Wong, Björn Petri, Paul Kubes

**Affiliations:** 1 Calvin, Phoebe & Joan Snyder Institute for Infection, Immunity & Inflammation, University of Calgary, Calgary, Alberta, Canada; 2 Department of Critical Care Medicine, University of Calgary, Calgary, Alberta, Canada; 3 Department of Physiology and Pharmacology, University of Calgary, Calgary, Alberta, Canada; Ludwig-Maximilians-Universität München, Germany

## Abstract

Platelets are central players in inflammation and are an important component of the innate immune response. The ability to visualize platelets within the live host is essential to understanding their role in these processes. Past approaches have involved adoptive transfer of labelled platelets, non-specific dyes, or the use of fluorescent antibodies to tag platelets *in vivo*. Often, these techniques result in either the activation of the platelet, or blockade of specific platelet receptors. In this report, we describe two new methods for intravital visualization of platelet biology, intravenous administration of labelled anti-CD49b, which labels all platelets, and CD41-YFP transgenic mice, in which a percentage of platelets express YFP. Both approaches label endogenous platelets and allow for their visualization using spinning-disk confocal fluorescent microscopy. Following LPS-induced inflammation, we were able to measure a significant increase in both the number and size of platelet aggregates observed within the vasculature of a number of different tissues. Real-time observation of these platelet aggregates reveals them to be large, dynamic structures that are continually expanding and sloughing-off into circulation. Using these techniques, we describe for the first time, platelet recruitment to, and behaviour within numerous tissues of the mouse, both under control conditions and following LPS induced inflammation.

## Introduction

The interface between innate and adaptive immunity is critically central to the host immune response. In recent years, platelets have emerged as an important component of this interface, shaping both the innate and adaptive immune responses [1]–[4]. Although primarily associated with hemostasis, platelets express a wide variety of immune receptors (TLRs, CD14, CD40, FcR) and release numerous immune effectors upon activation (IL1β, TGFβ, RANTES, MCP-1, CD40L) [1]. Together, these receptors and molecules allow platelets to modulate leukocyte recruitment [5], activation and effector functions such as phagocytosis, cytotoxicity and extracellular DNA release (NETs) [6], key events at the crossroads between innate and adaptive immunity. A summary from the Fourth Annual Platelet Colloquium concluded that platelets can recruit cells to sites of vascular injury, release pro- and anti-inflammatory factors, produce microparticles, and can induce thrombin generation [7]. These processes have been shown to be critical in the induction or progression of diseases such as atherosclerosis, sepsis, hepatitis, vascular restenosis, acute lung injury and transplant rejection.

Although some information about platelet localization can be gained from immunohistochemical staining of tissue sections or radioisotope labelling of adoptively transferred platelets [2], [8], the limitation of these techniques is the inability to study the dynamic interactions between platelets, leukocytes, and the vasculature. The development of intravital microscopy, and more recently, intravital spinning-disk confocal microscopy, has allowed for the observation of cellular behaviour within the tissue microenvironment of live animals. The capacity of spinning-disk confocal microscopy to capture individual frames of multiple fluorescence wavelengths in rapid succession, something that is not possible with conventional scanning-confocal microscopy, affords the ability to image rapidly moving particles, such as platelets, and makes this technology ideal for the study of platelet dynamics *in vivo*. The difficulty in studying platelets however, has been the identification of markers that can be used *in vivo* to specifically label platelets without affecting their behaviour.

Currently, most studies have utilized one of two general approaches; either intravenous administration of a non-specific fluorescent dye such as rhodamine 6-G [9], [10] or the adoptive transfer of platelets isolated from a donor animal, labelled *in vitro* with a fluorescent dye, and injected into a recipient animal [8], [11]. These techniques allow for the visualization of platelets within the live animal, and have provided insight into platelet recruitment and adherence; however, they too have limitations. Although rhodamine 6-G efficiently labels platelets *in vivo* and allows for visualization of platelet-vessel wall interactions, due to its non-specific nature it also results in the fluorescent labelling of all blood cells, including neutrophils and monocytes, key targets for platelet interactions. This makes it impossible to distinguish aggregates of platelets from leukocytes at sites of inflammation or to visualize platelets directly associated with rhodamine 6-G labelled leukocytes.

In contrast, *ex vivo* staining of purified platelets results in specific labelling and allows for the study of platelet interactions with other cells, however, only a small percentage of the platelets in the recipient animal are labelled following adoptive transfer (endogenous platelets remain unlabelled). This limits the ability to measure total platelet recruitment, aggregate size or to visualize platelet-platelet interactions. Additionally, platelets are easily activated by shear stress and temperature fluctuation [12], [13]. Thus one has to pay particular attention to the platelet isolation, labelling and transfusion to ensure that the adoptively transferred platelets are not activated and that their behaviour upon transfer mirrors that of endogenous platelets. Not trivial is the fact that such experiments require the killing of substantial numbers of additional animals in order to obtain platelets for transfer.

More recent approaches have focused on monoclonal antibody (mAb) labelling of platelets *in vivo*. Early attempts to use mAb against the pan-platelet marker CD41 were not optimal [14]. Although this approach allowed for the visualization of platelets *in vivo* [15], [16], mAb directed against CD41 have been reported to inhibit more than 80% of cellular adhesion to fibrinogen [17], an important molecule in platelet and cellular recruitment to sites of inflammation, and platelet aggregation [14], [18]. Other experiments have used an mAb directed against the platelet surface receptor GPIbβ [19], and although this mAb is able to label platelets *in vivo*, it blocks the ability of platelets to bind von Willebrand factor [20] and possibly Mac-1, important mediators of platelet adhesion and neutrophil-platelet interactions [21]. The undesired effects of anti-CD41 and anti-GPIbβ antibodies make these approaches to platelet labelling less than ideal for the study of platelet responses to inflammatory stimuli.

In our efforts to find a better surface marker to facilitate platelet labelling, without blocking platelet function, we have identified CD49b as a suitable target molecule. Fluorescently conjugated antibodies against this marker readily label platelets *in vivo* with no observable effect on platelet behaviour. Additionally, we have utilized a transgenic mouse expressing yellow fluorescent protein (YFP) under the control of the CD41 promoter [22] to further characterize platelet dynamics and to validate the results obtained by *in vivo* Ab labelling by CD49b. Finally, we highlight examples of how these approaches, combined with spinning-disk confocal microscopy and 3D reconstruction capabilities, reveal new and important data about platelet function *in vivo*.

## Results

### *In vivo* labelling of mouse platelets with anti-CD49b

**Figure 1:**
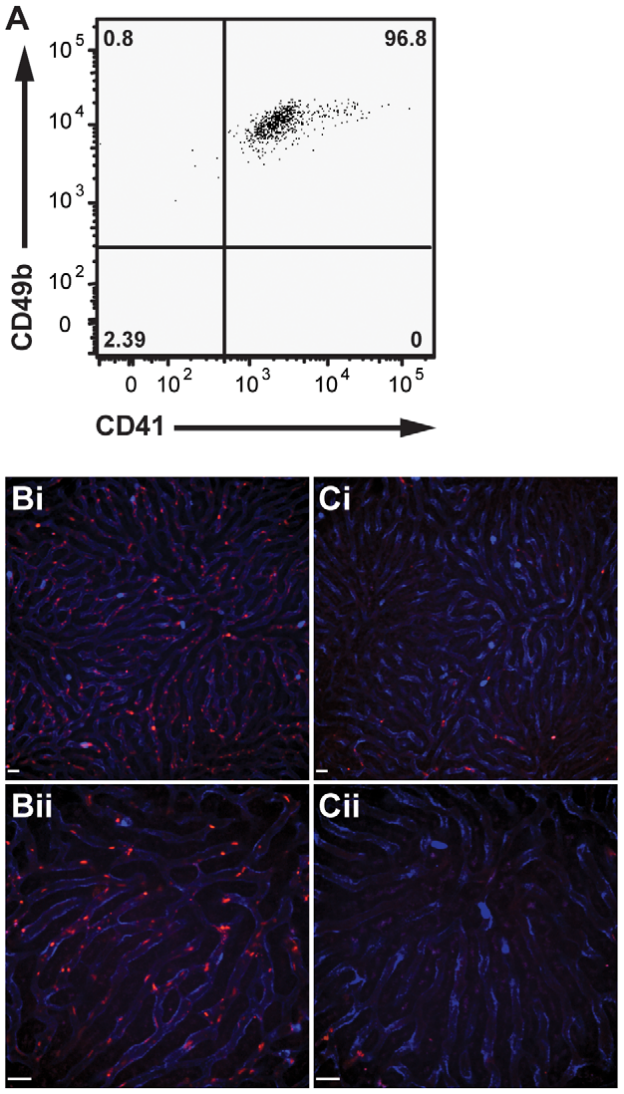
In vivo labelling of mouse platelets by intravenous injection of the anti-CD49b antibody HMα2. Anti-CD49b uniformly labels all CD41+ platelets in mouse blood (**A**). Intravenous injection of PE-conjugated anti-CD49b (red) labels circulating platelets within the mouse liver (**Bi** –10× objective, **Bii** –20× objective) and pre-treatment of mice with anti-thrombocyte serum (**Ci** –10× objective, **Cii** –20× objective) results in the loss of CD49b+ particles confirming particles labelled by CD49b are indeed platelets. Neutrophils labelled with Alexa Fluor 647-conjugated anti-Gr-1 (blue). All scale bars, 20 µm.

In blood, CD49b is expressed on NK cells, a subset of NKT cells and on platelets [23]–[25]. Due to this restricted expression, we decided to test the suitability of this molecule as a marker for platelets *in vivo*. Flow cytometric analysis of blood platelets demonstrates the mAb HMα2 directed against CD49b stains nearly all platelets as identified by the pan-platelet marker CD41 (**Fig. 1A**). This staining appears to be bright, stable and uniform, with a single population of CD49b+ platelets. These results identify this marker as a potential candidate for *in vivo* labelling of platelets for intravital microscopy applications. Intravenous (i.v.) administration of 1.6 µg of PE-conjugated anti-CD49b rapidly labelled a large number of particles within the liver vasculature that were several fold smaller than leukocytes (**Fig. 1Bi**, **Movie S1**). These particles were measured to have a diameter of 2.80±0.37 µm, which fall into the expected size range of mouse platelets [26]. Unexpectedly, many of these particles were observed to briefly interact with the sinusoid wall or with the few neutrophils present in an untreated liver. Although brief interactions could be observed, these particles almost never remained adherent but rather appear to freely circulate through the liver sinusoids (**Fig. 1Bii** **, Movie S2**).

To ensure the particle-endothelial interactions observed in control mice were not the result of surgical manipulation of the tissue, we examined particle behaviour within the ear microvasculature, a tissue that requires no surgical preparation prior to visualization of the blood vessels. Once again, no adherent, and a few interacting particles are seen under resting conditions (**Fig. S1, Movie S3**). These results indicate that the observed particle-endothelial interactions under basal conditions are independent of surgical manipulation of the tissue.

To further establish the identity of these particles as platelets, mice were pretreated with anti-thrombocyte serum for 20 h prior to intravital microscopy. In these platelet-depleted animals there was an absence of labelled particles following injection of PE-conjugated anti-CD49b (**Fig. 1Ci, Cii** **, Movie S4**). The lack of labelling in thrombocyte depleted mice, together with the flow cytometric analysis of CD41 and CD49b co-staining of mouse peripheral blood, positively identifies the labelled particles observed by intravital microscopy as platelets. Mice receiving intravenous anti-CD49b do not present any of the adverse health effects observed using anti-CD41 antibodies [14] and have remained stable for more than three hours, allowing for the intravital observation of platelets throughout this timeframe (unpublished observations).

It should be noted that this staining also labels NK cells within the tissue and blood (**Fig. S2**). These cells are extremely rare, fewer than 10/low power field of view in the mouse liver, despite this tissue being a main target and resident site for NK cells. They are much larger than a platelet, and their staining is fainter than that of the platelets, allowing for direct observation of platelet-NK cell interactions if needed. As such, these cells do not interfere with intravital platelet analysis. No *i*NKT cells were labelled with this approach. Treatment of Cxcr6^gfp/+^ mice, whose *i*NKT cells are GFP-positive, failed to result in dual labelling of these cells (data not shown).

### Use of anti-CD49b for the intravital study of platelet recruitment to the mouse liver

**Figure 2:**
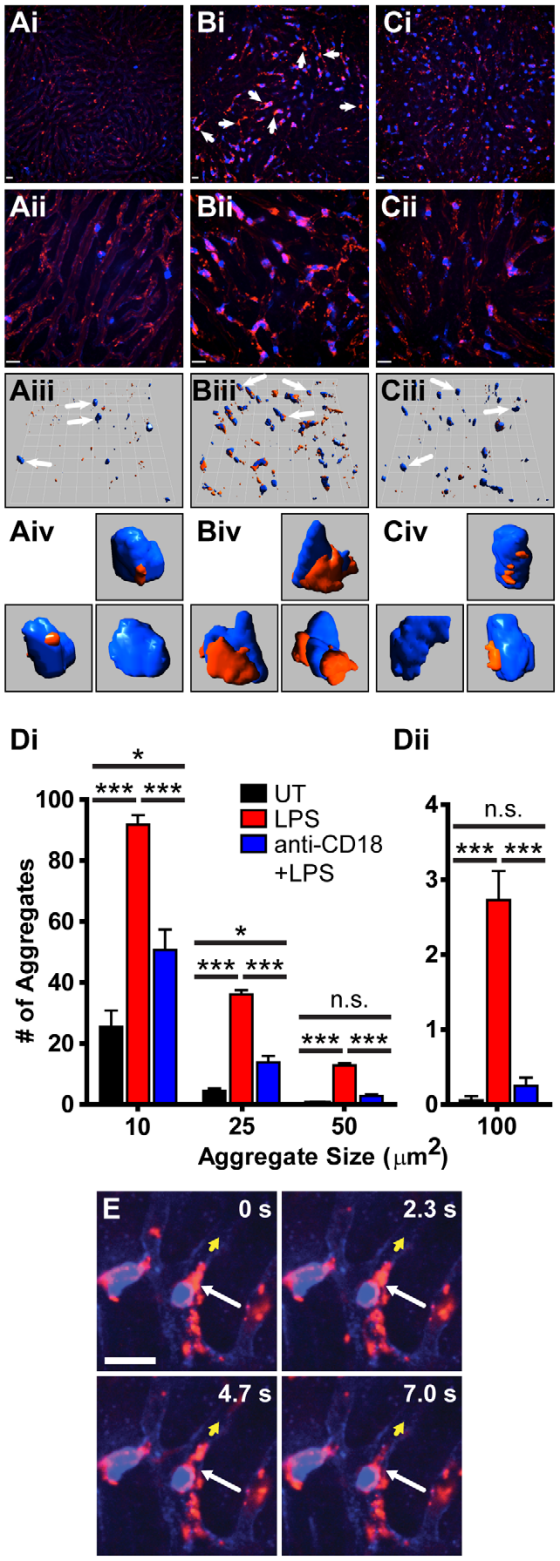
Quantification of platelet aggregation in the livers of mice treated with intravenous LPS using PE-conjugated anti-CD49b. Intravital microscopy of livers from untreated mice (**Ai–Aiv**), LPS treated mice (**Bi–Biv**) and mice pre-treated with an anti-CD18 blocking mAb followed by LPS (**Ci–Civ**). Representative fields of view at low power (10× objective) magnification (**Ai, Bi, Ci**) illustrating platelet aggregation in response to LPS and inhibition of aggregation following pre-treatment with an anti-CD18 blocking Ab. Platelets labelled with PE-conjugated anti-CD49b (red); neutrophils labelled with Alexa Fluor 647-conjugated anti-Gr-1 (blue). Arrows denote large platelet aggregates. Higher power (20× objective) extended focus images (**Aii, Bii, Cii**) and 3D opacity models (**Aiii, Biii, Ciii**) rendered from 29–30 z planes illustrating the extent of platelet aggregation in LPS treated mice. All scale bars, 20 µm; grid 25.7 µm. Select models of neutrophil-platelet aggregates (**Aiv, Biv, Civ**) extracted (as indicated by the white arrows) and enlarged from panel (iii). (**Di, Dii**) Quantification of the number of platelet aggregates equal to, or larger than the indicated sizes. *** *p*<0.001, * *p*<0.05, n.s. not significant (**E**) Time lapse images of neutrophil-platelet interactions illustrating the dynamic nature of the platelet aggregates within the liver. Long white arrow identifies a large platelet aggregate tearing off of an adherent neutrophil, short yellow arrow indicates direction of blood flow within the sinusoid. Sequential images represent 2.3 s intervals. Scale bar, 20 µm.

Treatment of mice with LPS has been reported to induce the recruitment of neutrophils and platelets to various tissues [6]. In sharp contrast to the liver of control animals (**Fig. 2Ai–iv**), intravital analysis of livers from mice treated with 1 mg/kg LPS i.v. for 4 h clearly demonstrates profound neutrophil recruitment (**Fig. 2Bi, Bii**). Furthermore, analysis of peripheral blood in these mice reveals a drop in the circulating platelet count (**Fig. S3**). Intravenous administration of PE-conjugated anti-CD49b allows, for the first time, the direct intravital visualization of endogenous platelet recruitment to this tissue. Platelets are readily observed to be interacting primarily with the large number of neutrophils already adherent within the liver. Many fewer interactions are seen between platelets and the liver sinusoids and almost no interaction between circulating neutrophils and adherent platelets can be observed. Many of the recruited platelets form aggregates, often the same size as, or larger than, the adherent neutrophils (**Fig. 2Bi** **, Movie S5**). Extended focus images generated from stacks of multiple xy planes at higher magnification reveals many of these aggregates are associated with neutrophils (**Fig. 2Bii**), forming from an initial adherence of platelets to a neutrophil followed by platelet-platelet interactions facilitating the expansion of the growing aggregate. Often, aggregates are seen to be spanning between two proximal neutrophils and appear to greatly reduce the blood flow in a number of liver sinusoids. By rendering 3D models of these z stacks with the Volocity software package we are able to visualize these platelet-neutrophil interactions from a number of different perspectives (**Fig. 2Biii** **, Movie S6**). Closer inspection of representative neutrophils from LPS treated mice illustrates the interaction between the large platelet aggregates and the adherent neutrophils (**Fig. 2 Biv**). These platelet aggregates cover large portions of the neutrophil surface and appear to “wrap around” the neutrophil. Through the generation of these z stacks, and the subsequent 3D images, we are able to determine these platelet aggregates are directly associated with the adherent neutrophils, an observation that would not be possible through the imaging of individual static sections. These findings are consistent with the observation that neutropenia precedes thrombocytopenia in this model of inflammation [2]. Unlike previous reports using other models of inflammation, we rarely see platelets first binding to endothelium followed by neutrophil recruitment to these adherent platelets [27]–[29].

In contrast, the few adherent neutrophils present in the liver of untreated mice are largely free of platelet interactions (**Fig. 2Aiii, Aiv**(representative 3D models)). In the few examples of neutrophil-platelet interactions in untreated mice, we do not see substantial platelet-platelet interactions and as such there is a failure to generate larger platelet aggregates. It is important to note our *in vivo* platelet labelling excludes platelet activation due to platelet isolation or *in vitro* labelling procedures.

Visualization of platelets within the mouse liver in response to LPS treatment allows for the characterization of receptors and adhesion molecules involved in platelet recruitment. Past reports have implicated the adhesion molecule CD18 in the binding of platelets to neutrophils [18], [30]–[32], dendritic cells [33], and T cells [34], although to date, the effect of CD18 blockade on platelet recruitment has not been visualized *in vivo*. As previously reported by our group [35], [36], pre-treatment of mice with a blocking Ab directed against CD18 has no effect on neutrophil recruitment to the liver in response to LPS (**Fig. 2Ci**). By contrast, CD18 blockade had a dramatic effect on platelet recruitment to the inflamed liver (**Fig. 2Ci–Civ**). Although individual platelets appeared to be recruited independent of CD18, large aggregates failed to form in mice treated with anti-CD18 blocking antibody.

Furthermore, because i.v. administration of anti-CD49b labels all platelets, it is possible to measure the size of the numerous platelet aggregates. The area of staining corresponding to each platelet aggregate was measured and the number of aggregates equal to, or greater than, specific size thresholds were enumerated. We observed a significant increase in both the total number of recruited platelets and the size of platelet aggregates within the livers of LPS treated mice (**Fig. 2Di, Dii**). This platelet recruitment and aggregation was significantly inhibited by the pre-treatment of mice with a blocking Ab against CD18.

The ability of CD18 blockade to inhibit platelet aggregation is particularly interesting given the fact that platelets have not been reported to express this molecule. One possible mechanism to explain this observation might involve the formation of microparticles. LPS treatment has been reported to result in the generation of neutrophil, endothelial and platelet derived microparticles. These microparticles are formed from the plasma membrane and can express cell surface markers and adhesion molecules derived from the cell from which they were released [37]–[39]. These particles have been reported to express CD18 and might facilitate platelet aggregation by bridging between ligands on individual platelets. Flow cytometric analysis of platelets from untreated and LPS-treated mice failed to detect any association between platelets and either neutrophil (GR1+) or Kupffer cell (F4/80+) derived membranes (**Fig. S4A, B**). Furthermore, expression of CD18 was not detected on platelets obtained from untreated or LPS-treated mice, further ruling out the association of platelets with CD18+ microparticles (**Fig. S4C**).

As we were unable to detect CD18 expression by platelets, it is most likely this molecule is expressed by other cells involved in platelet aggregation. In fact, CD18 is highly expressed by both neutrophils and Kupffer cells [40]–[43], and platelets express a number of ligands for β2 integrins (ICAM-2, Gp1bα, JAM-C) [30], [33]. These observations suggest CD18 expressing leukocytes within the liver vasculature bind platelets and initiate aggregation. In support of this model, depletion of either neutrophils or Kupffer cells dramatically inhibits platelet aggregation, indicating these cells are essential to the process (**Fig. S5**). Therefore, it is likely that circulating platelets must touch-down on, and anchor to, a neutrophil or Kupffer cell as a first step towards the generation of larger platelet aggregates.

Through the use of intravital microscopy we are able to visualize the behaviour of platelets within the liver sinusoids and their interactions with adherent neutrophils. It is clear from these studies that the platelet aggregates that form in response to LPS are not static entities, but rather, are dynamic structures, continually expanding and sloughing off (**Fig. 2E** **, Movie S7** (white arrow identifies a large platelet aggregate tearing off of a neutrophil)). These platelet aggregates often appear to “flow” across the surface of an adherent neutrophil (**Movie S6, S7**). In addition to platelet aggregates associated with adherent neutrophils, we are also able to observe circulating platelet-neutrophils aggregates (**Fig S6, Movie S8**). Neutrophils, covered with large platelet aggregates can be seen flowing through the liver sinusoids, with little interaction between the neutrophil and the vessel walls. These observations would not be possible with other techniques such as staining of tissue sections, intravital observation of adoptively transferred platelets, or *in vivo* labelling of platelets with rhodamine 6-G and are essential to our understanding of platelets in the host immune response.

### Use of CD41-YFP^ki/+^ mice for the intravital study of platelet recruitment to the mouse liver

The generation and characterization of CD41-YFP**^ki/+^** mice has been previously reported [22], although, to date these mice have not been used for the intravital study of platelet behaviour. In these mice, a percentage of platelets express YFP (**Fig. S7**) and thus are visible using fluorescence microscopy without the need to add an exogenous label. As such, these mice can be used to confirm that the platelet adhesion observed in Fig. 2 is independent of the labelling of platelets by anti-CD49b. As the percentage of YFP-labelled platelets varies significantly from mouse to mouse (from less than 10% to more than 40%) [22], all animals must be pre-screened by flow cytometry to ensure individuals in the control and treated groups have comparable numbers of labelled platelets. Mice used in this study had an average of 21.6%±5.9% YFP positive platelets.

**Figure 3:**
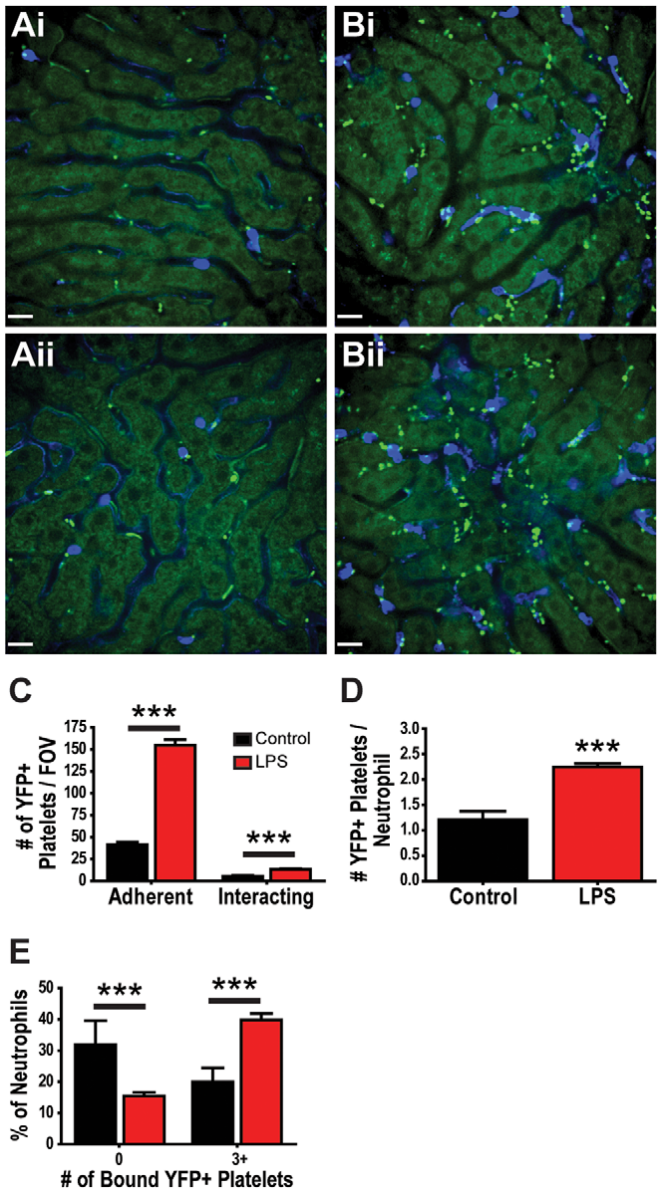
Quantification of platelet recruitment to livers of mice treated with intravenous LPS using CD41-YFP^ki/+^ mice. Representative fields of view of livers from untreated (**Ai, Aii**) and LPS treated (**Bi, Bii**) CD41-YFP**^ki/+^** mice. Neutrophils are labelled with Alexa Fluor 647-conjugated anti-Gr-1 (blue); YFP+ platelets and liver autofluorescence (green). All scale bars, 20 µm. (**C**) Quantification of the number of adherent (stationary for ≥30 s) and interacting (present in the field of view for but not stationary for ≥30 s) YFP+ platelets per field of view of liver in untreated and LPS treated CD41-YFP**^ki/+^** mice. (**D**) Average number of YFP+ platelets adherent to each neutrophil within the livers of untreated and LPS treated CD41-YFP**^ki/+^** mice. (**E**) Percentage of neutrophils with zero or with three or more adherent platelets within the livers of untreated and LPS treated CD41-YFP**^ki/+^** mice. *** *p*<0.001.

Intravital observation of YFP+ platelets in the liver sinusoids of untreated mice reveals little platelet adhesion or aggregation (**Fig. 3Ai, 3Aii**), an observation similar to what was seen when anti-CD49b was used to label the platelets. Some YFP+ platelets appear to interact with the sinusoidal walls or adherent neutrophils, touching down briefly, but then quickly releasing and re-entering the blood flow (**Movie S9**), a behaviour that is again consistent with that observed when platelets were labelled with anti-CD49b. In contrast, livers of mice treated with LPS demonstrate pronounced platelet recruitment (**Fig. 3Bi, 3Bii**), where YFP+ platelets are seen to be associated with the sinusoid walls and even more so with the numerous recruited neutrophils (**Movie S10**). It is important to note that only approximately 20% of platelets are visible in these mice and, as such, visualization of platelet-platelet interactions and platelet aggregation is limited. Furthermore, because not all platelets are labelled, it is impossible to measure the total size of platelet aggregates in this model. One advantage of these mice, however, is individual platelets can be visualized more easily, thus making it easier to study platelet behaviour on the level of the single platelet.

Quantification of the number of adherent (stationary for ≥30 s) and the number of interacting platelets (present in a field of view for ≥30 s but not stationary) demonstrates significant increases in LPS-treated mice when compared to untreated mice (**Fig. 3C**). Furthermore, we can characterize the interactions of individual platelets with adherent neutrophils in the liver. Following LPS treatment, the average number of YFP+ platelets bound by each neutrophil (platelets that remain adherent for ≥30 s) increased significantly as compared to untreated mice (**Fig. 3D**). Moreover, the percentage of neutrophils that were not associated with YFP+ platelets was significantly reduced and the percentage of neutrophils binding three or more YFP+ platelets increased significantly following treatment with LPS (**Fig. 3E**). These CD41-YFP**^ki/+^** data support the observations made using anti-CD49b where a 3-4 fold increase in the total number of platelet aggregates (≥10 µm^2^, **Fig. 2Di**), many of which are adherent to neutrophils, is seen in the livers of mice treated with LPS.

**Figure 4:**
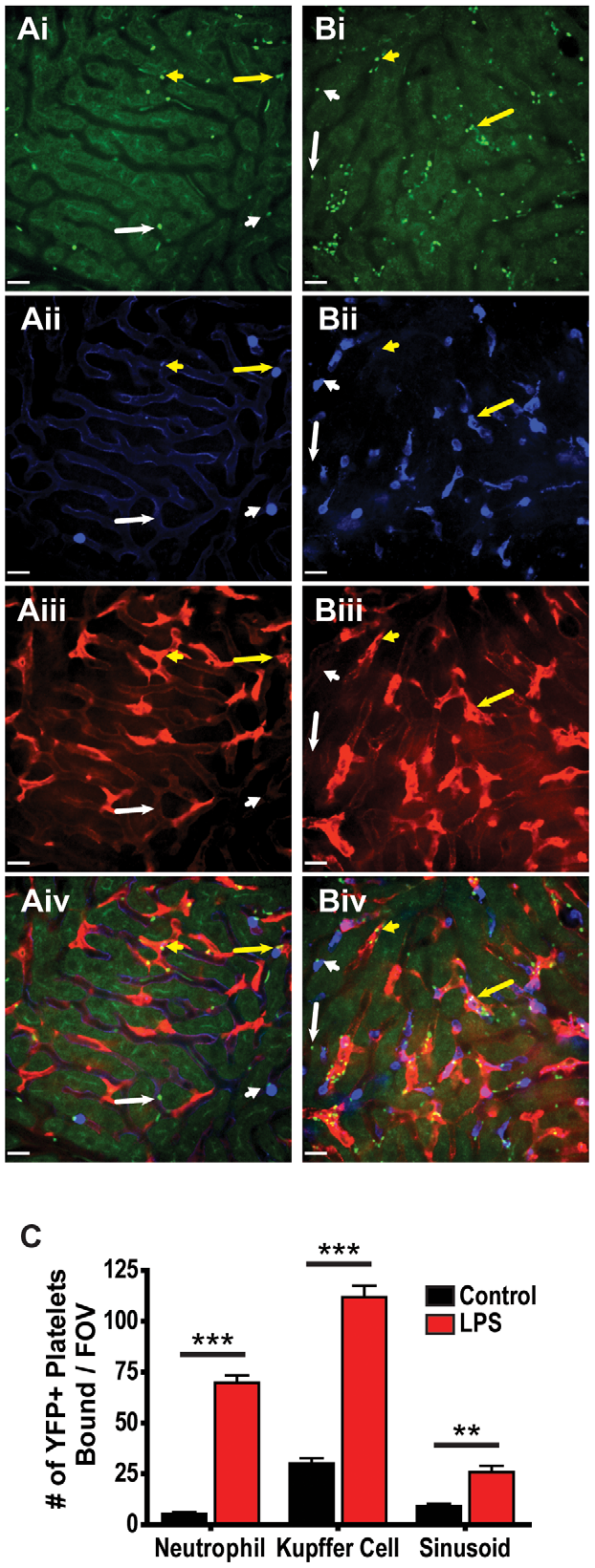
Characterization of platelet binding in the livers of mice treated with intravenous LPS using CD41-YFP^ki/+^ mice. Representative fields of view of livers from untreated (**A**) and LPS (**B**) treated CD41-YFP**^ki/+^** mice. Visualization of YFP+ platelets and liver autofluorescence (**Ai, Bi**), neutrophils labelled with Alexa Fluor 647-conjugated anti-Gr-1 (**Aii, Bii**), Kupffer cells labelled with PE-conjugated anti-F4/80 (**Aiii, Biii**), and overlay of fluorescent channels (**Aiv, Biv**). Examples of platelets interacting with the liver sinusoid away from neutrophils or Kupffer cells (long white arrows), platelets interacting with neutrophils (short white arrows), platelets interacting with Kupffer cells (short yellow arrows), and platelets appearing to interact with both Kupffer cells and neutrophils (long yellow arrows). All scale bars, 20 µm. (**C**) Quantification of the number of YFP+ platelets bound to neutrophils, Kupffer cells and liver sinusoids per field of view within livers of untreated and LPS treated CD41-YFP**^ki/+^** mice. *** *p*<0.001, ** *p*<0.01.

A more detailed understanding of platelet recruitment to the liver following LPS exposure can be obtained through the use of additional stains to identify other cell populations. Intravenous injection of PE-conjugated anti-F4/80 rapidly labels Kupffer cells, the liver resident macrophage population (**Fig. 4A, B**), and allows for visualization of platelet interactions with these cells. In livers of LPS treated CD41-YFP**^ki/+^** mice co-stained for neutrophils and Kupffer cells, platelets can be seen to adhere to neutrophils, Kupffer cells, regions of the hepatic sinusoids devoid of neutrophils and Kupffer cells, and to areas of co-localization of neutrophils and Kupffer cells, suggesting no single cell type is responsible for all platelet adhesion in the liver (**Fig. 4Aiv, Biv** **, Movie S11**). Enumeration of bound platelets demonstrates a significant increase in adherence to neutrophils, Kupffer cells, and to liver sinusoidal endothelium following LPS exposure (**Fig. 4C**). Interestingly, a far greater number of platelets are found to be associated with Kupffer cells than are seen associated with neutrophils, suggesting that Kupffer cells play a central role in platelet aggregation within the liver, an observation to our knowledge never previously noted. Although this finding is novel, it is not entirely surprising given that Kupffer cells occupy a greater area than do neutrophils in any given field of view (**Fig. S8**), and as such have the capacity to recruit a greater number of platelets.

Taken together, the data generated in the CD41-YFP**^ki/+^** mice support the observations made using anti-CD49b to visualize platelets and validate this molecule for *in vivo* labelling of the total peripheral platelet population in mice. Both approaches for visualizing platelets resulted in an approximately 4-fold increase in platelet adhesion (≥10 µm^2^, **Fig. 2Di** **,** **3C**) with many of the recruited platelets appearing to be associated with adherent neutrophils within the liver. Furthermore, as platelet recruitment to the liver was comparable between the two models, one can conclude that labelling with anti-CD49b neither blocks, nor enhances platelet recruitment to the liver in response to LPS challenge and allows for the observation of normal platelet behaviour *in vivo*.

### Visualization of platelet recruitment in other tissues using anti-CD49b

**Figure 5:**
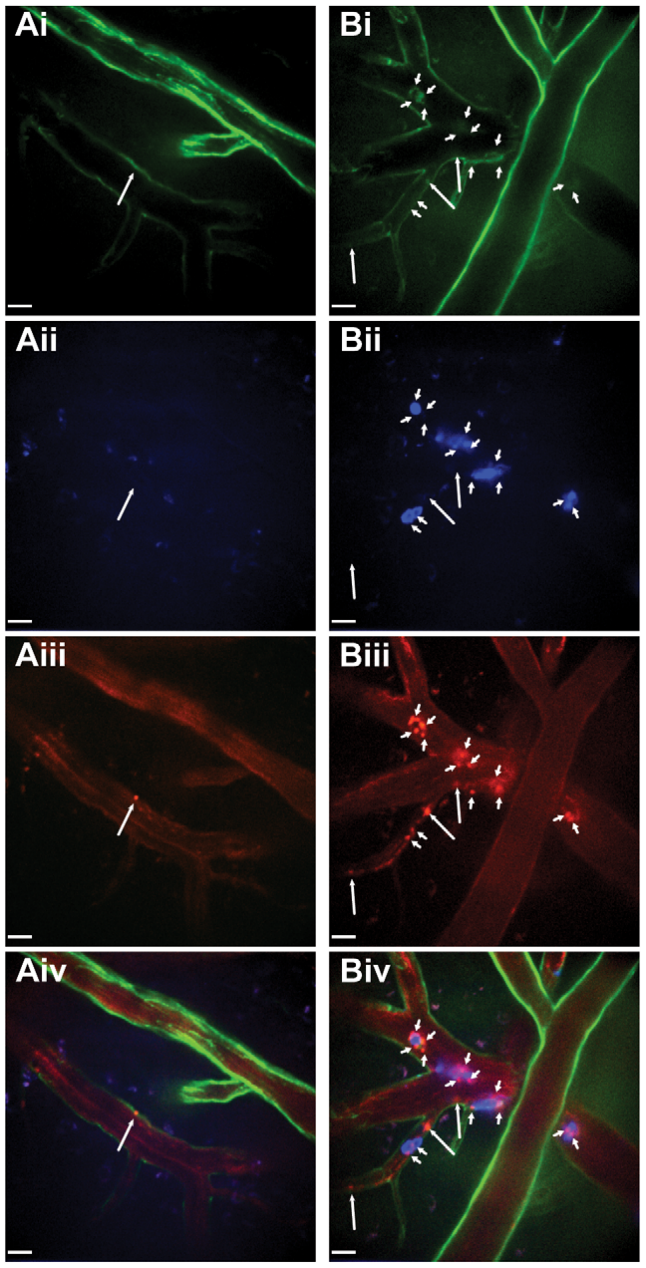
Intravital visualization of platelets labelled with PE-conjugated anti-CD49b within the mouse brain. Spinning-disk confocal-microscopic imaging of brain pial arterioles and postcapillary venules in untreated (**A**) and LPS treated mice (**B**). Visualization of endothelium with Alexa Fluor 488-conjugated anti-CD31 (**Ai, Bi**), neutrophils labelled with Alexa Fluor 647-conjugated anti-Gr-1 (**Aii, Bii**), platelets stained with PE-conjugated anti-CD49b (**Aiii, Biii**), and multi-channel overlay (**Aiv, Biv**). There was a marked absence of platelet or neutrophil adhesion in brain pial vessels of untreated mice (**A**), however, profound neutrophil and platelet recruitment within brain venules (faint CD31 staining), but not in brain pial arterioles (bright CD31 staining), following LPS treatment (**B**). Small arrows identify platelets interacting with neutrophils; large arrows identify platelets interacting with the endothelium. All scale bars, 20 µm.

We have demonstrated the novel use of PE-conjugated anti-CD49b to readily visualize platelet dynamics in the mouse liver; however, this technique can also be applied to other tissues. Intravital visualization of the mouse brain microvasculature using anti-PECAM-1 reveals no platelet aggregation, and rare platelet-endothelial interactions under resting conditions (**Fig. 5A** **, Movie S12**).

In contrast to untreated mice, neutrophil and platelet recruitment can be readily observed in the brain 4 h following LPS treatment (**Fig. 5B** **, Movie S13**). Platelets can be seen adhering to primarily to adherent neutrophils with a few interactions with the vascular endothelium. Almost all of these interactions occur in the postcapillary venules of the brain with little recruitment seen in brain arterioles. Similar to what was observed in the liver, these platelet interactions appear to be very dynamic, with platelets adhering to neutrophils or endothelium and forming unstable aggregates that, after a period of time, slough off into the circulation.

**Figure 6:**
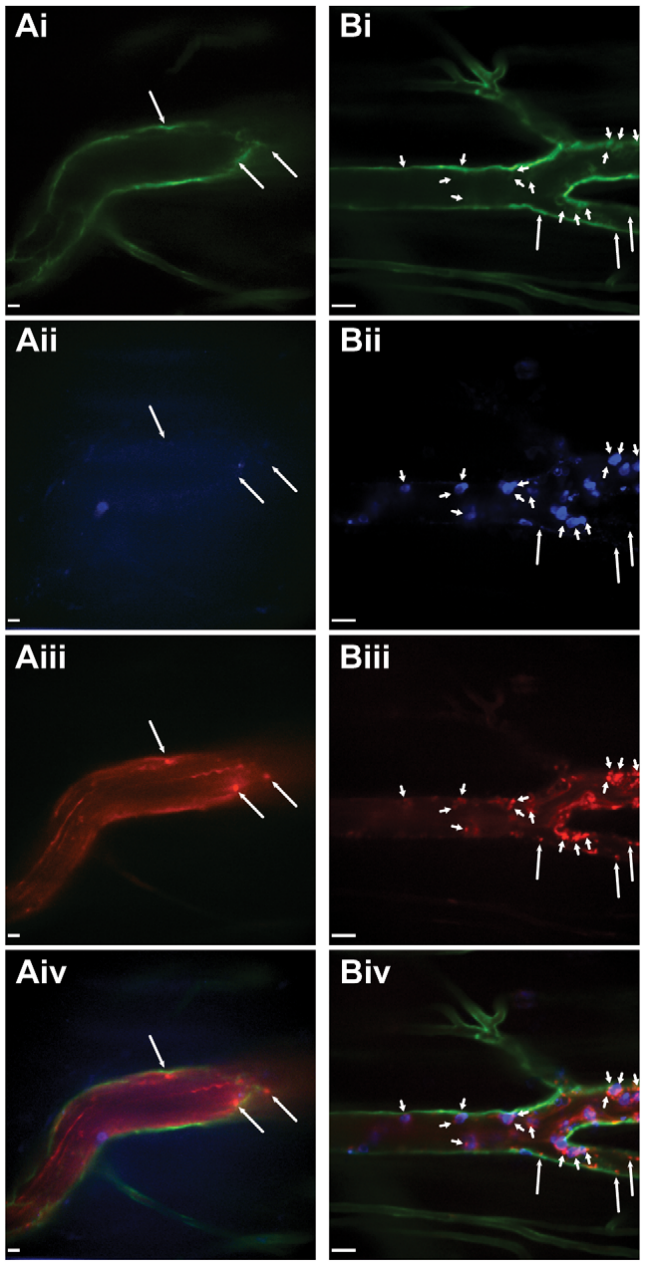
Intravital visualization of platelets labelled with PE-conjugated anti-CD49b within the mouse cremaster muscle. Spinning-disk confocal-microscopic imaging of cremaster postcapillary venules in untreated (**A**) and LPS treated mice (**B**). Visualization of endothelium with Alexa Fluor 488-conjugated anti-CD31 (**Ai, Bi**), neutrophils labelled with Alexa Fluor 647-conjugated anti-Gr-1 (**Aii, Bii**), platelets stained with PE-conjugated anti-CD49b (**Aiii, Biii**), and multi-channel overlay (**Aiv, Biv**). A small number of platelets are seen to interact with the postcapillary endothelium in the cremaster muscle under basal conditions (**A**). Neutrophil recruitment and platelet interactions increase dramatically in the vessels of the cremaster muscle following LPS treatment (**B**). Small arrows identify platelets interacting with neutrophils; large arrows identify platelets interacting with the endothelium. All scale bars, 20 µm.

Platelets can also be observed in muscle microvasculature using PE-conjugated anti-CD49b. Intravital visualization of postcapillary venules in the cremaster muscle using anti-PECAM-1 reveals little platelet adherence in untreated animals, although some platelets can be seen interacting with the vascular endothelium, appearing to touch-down and then quickly release (**Fig. 6A** **, Movie S14**). Following LPS exposure, however, dramatic neutrophil and platelet recruitment can be observed (**Fig. 6B** **, Movie S15**). Again, platelets are seen to interact with, and adhere to, both the vessel endothelium and neutrophils in a dynamic fashion. These videos also demonstrate that platelet interaction with neutrophils does not require the neutrophil to be adherent. In some instances, neutrophils covered in platelets can be observed in circulation and seen rolling on the cremaster vascular endothelium.

## Discussion

Platelets are emerging as central players in both innate and adaptive immunity [1], [3], [4], [6]. As such, a more in depth knowledge of their role in inflammation, immune cell recruitment and cellular activation is critical to our understanding of these processes. Intravital microscopy can reveal elegant details about the dynamic interactions between platelets and their surrounding environment, information that is not possible to obtain from other techniques. Although recent advances have been made in the ability to label and visualize platelets in the living animal, isolation of platelets for labelling or injection of mAb against specific platelet receptors such as CD41 have a number of limitations.

Intravenous administration of anti-CD49b rapidly labels the total peripheral platelet population and can facilitate the visualization of platelets by intravital microscopy. The use of the anti-CD49b mAb HMα2 for the labelling of platelets has several advantages over previous approaches. First, i.v. administration of anti-CD49b, unlike CD41 [14], does not induce an adverse systemic response. Second, HMα2 is widely available and can be ordered pre-conjugated to a number of different fluorophores. Third, this technique can be used to visualize platelets in mice with various genetic backgrounds and gene knockouts whereas models such as CD41-YFP**^ki/+^** mice need to be bred onto other genetic backgrounds. Finally, HMα2 does not interfere with the ability of platelets to bind fibrinogen [44], an important property if one is to examine the role of platelets in inflammation. Using this technique, we have been able to study *in vivo*, as one example, the formation of platelet aggregates in response to LPS exposure. Furthermore, as all platelets are labelled using this procedure, it is possible to measure the size of individual platelet aggregates and to visualize platelet-platelet interactions. These observations are not possible if only a fraction of platelets are labelled, as is the case in experiments using adoptive transfer of labelled platelets.

In addition to visualizing platelets with mAb, we have utilized the CD41-YFP**^ki/+^** mouse [22] to validate our approach of labelling platelets with anti-CD49b. The behaviour of platelets in LPS treated CD41-YFP**^ki/+^** mice, a model where no exogenous label is added to the platelets, parallels that observed with anti-CD49b in LPS treated C57BL/6 mice. As a result we have been able to confirm that labelling of platelets with anti-CD49b does not alter their response to an inflammatory stimulus and validates the use of this mAb for the visualization of platelet dynamics *in vivo*. In CD41-YFP**^ki/+^** mice, only a percentage of platelets express YFP and thus are visible by intravital fluorescence microscopy. Whereas it is not possible to measure the size of a given platelet aggregate in this model, it is possible to observe individual platelets within aggregates. This allows for study of individual platelet behaviour and adhesion, even in areas of massive inflammation. Through the use of additional *in vivo* labels, such as anti-Gr-1 and anti-F4/80, we are able to observe and track the interactions between platelets, neutrophils and Kupffer cells within the LPS challenged liver. Interestingly, Kupffer cells bound the most platelets although the purpose platelet adherence to these cells remains to be determined.

Using these two techniques, we have for the first time, observed *in vivo* platelets under both control, and inflammatory conditions. From these experiments we have identified several novel aspects of platelet behaviour. In some tissues, such as the liver, platelets are observed to frequently interact with the blood vessel walls under basal conditions, briefly touching down and then releasing back into circulation, appearing to probe or patrol the unactivated endothelium. In contrast, other tissues, such as the brain, demonstrate much less frequent platelet-endothelial cell interactions under control conditions. Following LPS stimulation, we observe large numbers of platelets interacting with adherent neutrophils, appearing to both roll across the surface of the neutrophil and to firmly adhere to these cells. Platelets are also observed to directly interact with other cell types, such as Kupffer cells and endothelial cells. In contrast to previous reports, few neutrophils are observed being recruited to adherent platelets. Furthermore, following LPS stimulation, platelets are observed to form large, dynamic aggregates that are continually expanding and sloughing off into circulation. These aggregates are dependent on CD18-mediated adhesion and blockade of this integrin completely disrupts the formation of these structures. Finally, in addition to interactions with adherent cells, platelets were also observed to interact with circulating neutrophils following LPS treatment. These circulating platelet-neutrophil aggregates are similar to platelet-leukocyte aggregates (PLA) frequently found in the blood of septic patients [45]–[48], and are thought to contribute to disease pathogenesis, potentially through the occlusion of organ microvasculature. This work represents the first *in vivo* observation of these platelet-neutrophil aggregates and future studies will be aimed at understanding the formation and role of these aggregates in severe inflammatory disease.

Through the use of multiple techniques for platelet labelling, we report that anti-CD49b provides an accurate and comprehensive picture of platelet dynamics in a variety of tissues. We were able to measure the number and size of platelet aggregates, to track individual platelet behaviour, and to study the numerous interactions between a given platelet and its surrounding environment. Although anti-CD49b may have some effect on platelet binding to collagen, and as such may not be appropriate for some models of platelet recruitment, the ability to label and visualize platelets *in vivo* using this antibody, without affecting the ability of platelets to bind important mediators such as fibrinogen or von Willebrand factor, affords many new opportunities for the study of cellular recruitment to sites of inflammation, platelet interactions with bacteria or parasites, platelet-neutrophil-endothelium interactions, and the role of platelets in modulating cellular activation, such as the production of NETs by neutrophils [6]. These new experimental possibilities will greatly expand our knowledge of these processes and will help facilitate the development of novel treatment approaches and strategies for numerous conditions involving platelet activation or dysfunction.

## Methods

### Ethics Statement

All experiments involving animals were approved by the University of Calgary Animal Care Committee (Protocol # MO8131) and conform to the guidelines established by the Canadian Council for Animal Care. For all experiments, mice were anesthetised by intra-peritoneal (i.p.) injection of 200 mg/kg ketamine (Bayer Inc Animal Health, Toronto, Ontario, Canada) and 10 mg/kg xylazine (Bimeda-MTC, Cambridge, Ontario, Canada).

### Mice

Wildtype C57BL/6 (The Jackson Laboratory, Bar Harbor, ME) and CD41-YFP**^ki/+^** (a generous gift from Dr. Kelly McNagny, University of British Columbia, Vancouver, British Columbia, Canada) colonies were maintained in specific-pathogen free facilities at the University of Calgary. At the time of use, animals were between 7 and 10 weeks of age and weighed 20–30 g.

### Antibodies and Treatments

Phycoerythrin (PE)-conjugated and allophycocyanin (APC)-conjugated Armenian hamster anti-mouse CD49b (clone HMa2), PE-conjugated rat anti-mouse CD41 (clone MWReg30), and FITC-conjugated goat-anti-rat IgG were purchased from BD Biosciences Pharmingen (San Diego, CA). PE-conjugated rat anti-mouse F4/80 (clone BM8), unconjugated and Alexa Fluor 647-conjugated rat anti-mouse Ly-6G (GR1) (clone RB6-8C5), unconjugated rat anti-mouse CD18 (clone GAME-46), unconjugated rat anti-mouse PECAM-1 (clone 390), and unconjugated rat IgG1 isotype control antibodies were purchased from eBioscience (San Diego, CA). For intravital microscopy, rat anti-mouse PECAM-1 mAb was conjugated to Alexa Fluor 488 using a protein labelling kit as per the manufacturer's instructions (Invitrogen, Eugene, OR). *In vivo* blockade of CD18 was achieved by i.v. administration of 100 µg of anti-CD18 mAb 20 min prior to LPS treatment. Platelet depletion was performed by i.p. injection of 100 µl of anti-thrombocyte serum (Cedarlane, Burlington, Ontario, Canada). Highly purified lipopolysaccharide (LPS) (Escherichia coli O111∶B4) was purchased from Calbiochem (EMD Sciences, San Diego, CA). For liver and brain studies, mice were treated with 1 mg/kg LPS i.v. 4 h prior to intravital analysis; in cremaster studies, mice received 0.5 µg/kg LPS in 200 µl of saline injected intrascrotally 4 h prior to visualization. Neutrophil depletion was performed by i.p. injection of 200 µg of unconjugated GR-1 24 h prior to LPS treatment. Kupffer cell depletion was achieved by i.v. injection of 200 µl of clodronate liposomes 30 h prior to LPS treatment as previously described [49], [50].

### Flow Cytometry

Approximately 900 µl of blood was collected from anesthetised mice via cardiac puncture into a syringe containing 100 µl of acid-citrate dextrose. For platelets, blood was centrifuged for 10 min at 100×*g* at room temperature in a desktop microfuge and the platelet rich plasma was collected. Collected plasma was centrifuged a second time at 100×*g* for 3 min to remove any contaminating red blood cells. Platelet rich plasma was collected into a new microfuge tube and stained with mAb diluted in FACS wash buffer (PBS containing 1 mM EDTA, 2% FBS) for 30 min on ice. Platelets were washed with cold FACS wash buffer, pelleted by centrifugation, resuspended in 100 µl of cold FACS wash buffer. For leukocytes, whole blood was lysed in a 3× volume of ACK Lysing Buffer (Lonza, Walkersville, MD), pelleted by centrifugation, washed with cold FACS wash buffer. Cells were stained with mAb diluted in FACS wash buffer for 30 min on ice, washed and resuspended in cold FACS wash buffer. Analysis was performed on either a FACScan (Beckton Dickinson, Mississauga, Ontario, Canada) or Attune Acoustic Focusing Cytometer (Life Technologies, Carlsbad, California).

### Platelet Counts

Approximately 450 µl of blood was collected from anesthetised animals via cardiac puncture into a syringe containing 50 µl of 0.5 M EDTA. Blood was transferred to EDTA coated microtainer tubes (Beckton Dickinson). Samples were transported to Calgary Lab Services for haematological analysis.

### Spinning-disk Confocal Intravital Microscopy (IVM)

Two different spinning-disk confocal microscopes were used in these studies.

Liver microscopy was performed using an Olympus IX81 inverted microscope (Olympus, Center Valley, PA), equipped with an Olympus focus drive and a motorized stage (Applied Scientific Instrumentation, Eugene, OR). This microscope is fitted with a motorized objective turret equipped with UPLANSAPO 10×/0.40 and UPLANSAPO 20×/0.70 objective lenses and is mounted to an optical table (Newport, Irvine, CA) to minimize vibration when imaging. This microscope is coupled to a confocal light path (WaveFx; Quorum Technologies, Guelph, Ontario, Canada) based on a modified Yokogawa CSU-10 head (Yokogawa Electric Corporation, Tokyo, Japan).

Brain, cremaster, and ear microscopy was performed using an Olympus BX51WI upright microscope (Olympus), equipped with a Ludl focus drive and a motorized stage (Applied Scientific Instrumentation). This microscope is mounted to a optical breadboard (Newport) to minimize vibration, fitted with UPLANFL N 10×/0.30W and XLUMPPlanFI 20×/0.95W objective lenses, and is coupled to a confocal light path (WaveFx; Quorum Technologies) based on a modified Yokogawa CSU-10 head (Yokogawa Electric Corporation).

For both microscopes, each of 491-, 561-, and 642-nm excitation laser wavelengths (Cobolt, Stockholm, Sweden) were sequentially controlled and merged into a single optic cable using an LMM5 laser merge module (Spectral Applied Research, Richmond Hill, Ontario, Canada). Fluorescence was visualized through one of ET 525/50M (green channel), FF 593/40 (red channel), or ET 700/75M (far red channel) band pass emission filters (Semrock, Rochester, NY) driven by a MAC 6000 Modular Automation Controller (Ludi Electronic Products, Ltd., Hawthorne, NY) and detected with a 512×512 pixels back-thinned EMCCD camera (C9100-13, Hamamatsu, Bridgewater, NJ). Volocity Acquisition software (V5.2.1 – Inverted; V4.3.2 – Upright) (Improvision Inc., Lexington, MA) was used to drive the confocal microscope. Image acquisition settings varied according to the microscope used and the tissue visualized. Typical laser power, exposure time and sensitivity settings are as follows, Liver (inverted); green channel (autofluorescence –80%, 415 ms, 215; CD41-YFP –75%, 425 ms, 250), red channel (CD49b –90%, 150 ms, 210; F4/80–90%, 100 ms, 210), far red channel (Ly-6G –80%, 300 ms, 200), Brain (upright); green channel (PECAM-1–95%, 300 ms, 240), red channel (CD49b –100%, 70 ms, 240), far red channel (Ly-6C –100%, 300 ms, 250), Cremaster and Ear (upright); green channel (PECAM-1–80%, 500 ms, 115), red channel (CD49b –95%, 500 ms, 120), far red channel (Ly-6G –80%, 500 ms, 120). Green, red, and far red channels were overlaid using brightest point settings before exporting in .tiff or .avi format.

### Preparation of the Mouse Liver for Intravital Microscopy

Intravital microscopy of the mouse liver was performed as previously described [51]. Briefly, the tail vein of anesthetised mice was cannulated to permit the delivery of fluorescently labelled Ab and for maintenance of anesthetic. Mouse body temperature was maintained using an infrared heat lamp. A midline incision followed by a lateral incision along the costal margin to the midaxillary line was performed to expose the liver. The mouse was place in a right lateral position and the ligaments attaching the liver to the diaphragm and the stomach were cut allowing the liver to be externalized onto a glass coverslip on the inverted microscope stage. Exposed abdominal tissues were covered with saline-soaked gauze to prevent dehydration. The liver was draped with a saline soaked KimWipe to avoid tissue dehydration and to help restrict movement of the tissue on the slide.

### Preparation of the mouse brain for IVM

The tail vein of an anesthetised mouse was cannulated to permit intravenous delivery of antibodies and additional anesthetic, if required. To isolate movement, the animal's head was held in a stereotaxic board. Skin covering the parietal bone of the mouse skull was reflected and the left parietal bone was carefully thinned using a high-speed drill (Fine Science Tools, North Vancouver, British Columbia, Canada) to allow for transillumination. Care was taken as to not open the cranial vault to ensure physiological pressures and blood flow were maintained. Intravital visualization of leukocyte biology within the pial microvasculature was performed through this thinned skull tissue using an upright microscope.

### Preparation of the mouse cremaster for IVM

The mouse cremaster muscle was used to study neutrophil recruitment as previously described [52]. In brief, the jugular vein of an anesthetised mouse was cannulated to permit intravenous delivery of antibodies and additional anaesthetic, if required. Mice were placed on a special cremaster preparation board and body temperature was maintained through a heat pad. An incision was made in the scrotal skin to expose the left cremaster muscle, which was then carefully dissected free of the associated fascia. The cremaster muscle was cut longitudinally with a cautery. The testicle and the epididymis were separated from the underlying muscle and were moved into the abdominal cavity. The muscle was held flat on an optically clear viewing pedestal and was secured along the edges with 4–0 suture. The exposed tissue was superfused with 37°C warmed bicarbonate-buffered saline, pH 7.4 and covered with a coverslip. Intravital visualization of leukocyte biology was performed using an upright microscope.

### Preparation of the mouse ear for IVM

The jugular vein of an anesthetised mouse was cannulated to permit intravenous delivery of antibodies and additional anaesthetic, if required. The mouse was placed on a heat pad to maintain the body temperature and the dorsal hair from the right ear was gently removed using depilatory cream (Nair; Church & Dwight Co., Inc, Princeton, NJ) without causing any irritation. The ear was carefully flattened out on an elevated preparation board, superfused with 37°C warmed bicarbonate-buffered saline, pH 7.4, and held in place with a coverslip applied on the dorsal side of the ear. Intravital visualization of leukocyte biology was performed using an upright microscope.

### Image Processing

Still images were exported from the Volocity (Improvision) acquisition software as .tif images. Images for platelet aggregate quantification were imported directly into ImageJ. Display items were processed using Photoshop (Adobe, San Jose, CA) to adjust the minimum threshold values for each of the fluorescence channels. The same threshold values were applied to images from all treatment groups within a single experiment. Videos underwent contrast enhancement within the Volocity software package, adjusting the Black Point for each fluorescence channel. Again, the same settings were applied to the videos of all treatment groups within a given experiment. Videos were exported as .avi files and were converted to an appropriate size, resolution, and frame rate using Microsoft Movie Maker (Microsoft Canada, Mississauga, Ontario, Canada) and Prism Video Converter Software (NCH Software Inc., Greenwood Village CO).

### 3D Model Generation

Z Stacks of xy planes (0.5 µm intervals) were imaged using an inverted spinning-disk confocal microscope using either the ASI focus drive (Applied Scientific Instrumentation) or Olympus focus drive (Olympus). 3D isosurface models of platelet aggregates were rendered within the Volocity software package (Improvision) using the 3D-opacity option. The same Black Level settings for each fluorescence channel were applied to all images. For 3D videos, individual frames were rendered using the default photobleaching compensation function within the Volocity software package.

### Semi-quantitative Analysis of Platelet Aggregates

Snapshots were generated from intravital videos and the images corresponding to the red fluorescence channel alone (PE-conjugated anti-CD49b) were exported as .tif documents. For analysis of aggregate size and number, images were opened with the ImageJ software package V1.41 (NIH at http://rsb.info.nih.gov/ij/) and image contrast was set to maximum to sharply define the borders of each platelet aggregate. To account for variability in background fluorescence between experiments and between Ab lot, and to eliminate fluorescence attributed to rapidly circulating platelets, the minimum brightness threshold was adjusted for each image. To determine the threshold value, LPS treated mice were included in all experiments. Threshold values were set to yield an average of 12–14 aggregates ≥50 µm^2^ for all fields of view of LPS treated animals. This number was empirically determined through examination of fields of view from LPS treated animals and enumeration of platelet aggregates visualized to be the size of, or larger than neutrophils. Once the threshold value was determined for each experiment, this value was then applied to images from all treatment groups within the experiment, thereby allowing for direct comparison of aggregate sizes and number between treatment groups. Once the minimum brightness threshold was set for each image, the number of platelet aggregates ≥10, 25, 50, or 100 µm^2^ were counted using the Analyze Particles function within ImageJ.

### Statistical Analysis

The number of platelet aggregates within each size category (Fig. 2D, S5) was compared between treatment groups using a one-way analysis of variance (ANOVA) with a Bonferroni post-test for multi-group comparison. Untreated (n = 3), LPS treated (n = 5), anti-CD18 + LPS treated (n = 4), neutrophil depleted + LPS (n = 5), Kupffer cell depleted + LPS (n = 4) neutrophil and Kupffer cell depleted + LPS (n = 3); a minimum of 5 fields of view were analyzed for each animal.

Platelet counts (Fig. S2) between untreated and LPS treated mice were compared using an unpaired Student's t-test. Untreated (n = 5), LPS treated (n = 4).

Comparisons of the number of adherent platelets, interacting platelets (Fig. 3C), and platelets bound per neutrophil (Fig. 3D) between untreated and LPS-treated mice were made using unpaired Student's t-tests. Comparison of the percentage of neutrophils with either 0 or 3 platelets bound (Fig. 3E) in the untreated and LPS treated groups was made using a two-way ANOVA with a Bonferroni post-test for multi-group comparison. Untreated (n = 4), LPS treated (n = 5); a minimum of 4 fields of view were analyzed for each animal.

Comparison of the number of platelets bound to neutrophils, KC, and sinusoids (Fig. 4C) between untreated and LPS treated mice was made using a two-way ANOVA with a Bonferroni post-test for multi-group comparison. Untreated (n = 4), LPS treated (n = 5); a minimum of 4 fields of view were analyzed for each animal.

Comparisons in the total area of neutrophil and Kupffer cell staining (Fig. S7) between untreated and LPS treated mice were made using a two-way ANOVA with a Bonferroni post-test for multi-group comparison. Area of staining was measured in 4 mice in each treatment group (2–4 FOV/mouse).

## Supporting Information

Figure S1 **Intravital visualization of platelets labelled with PE-conjugated anti-CD49b within the mouse ear.** Spinning-disk confocal-microscopic imaging of mouse ear microvasculature in untreated mice. Visualization of endothelium with Alexa Fluor 488-conjugated anti-CD31 (**i**), neutrophils labelled with Alexa Fluor 647-conjugated anti-Gr-1 (**ii**), platelets stained with PE-conjugated anti-CD49b (**iii**), and multi-channel overlay (**iv**). No adherent and few endothelial-interacting platelets were seen in the mouse ear, a tissue visualized without surgical intervention, under control conditions. All scale bars, 20 µm.
(TIF)

Figure S2 **In vivo labelling of mouse NK cells by intravenous injection of the anti-CD49b antibody HMα2.** Intravenous administered PE-conjugated anti-CD49b (red) also labels NK cells *in vivo* (white arrow). Their larger size and less intense staining makes labelled NK cells easily discernable from nearby platelets (small yellow arrows). Neutrophils are labelled with Alexa Fluor 647-conjugated anti-Gr-1 (blue); green is liver autofluorescence to illustrate vessels. Scale bar, 20 µm.
(TIF)

Figure S3 **Intravenous treatment with LPS results in a reduction in circulating platelets.** Circulating platelet counts from untreated and mice treated with 1 mg/kg LPS intravenously for 4 h. ** *p* = 0.01.
(TIF)

Figure S4 **Flow cytometric analysis of platelets from untreated and LPS-treated mice.** Absence of neutrophil associated (GR1) (**A**) or macrophage associated (F4/80) (**B**) surface markers on platelets from untreated or LPS-treated mice. Staining for CD18 expression on platelets (**C, left panel**) and granulocytes (**D, right panel**) obtained from untreated or LPS-treated mice.
(TIF)

Figure S5 **Effect of neutrophil and Kupffer cell depletion on platelet aggregation in the mouse liver in response to LPS treatment.** Quantification of the number of platelet aggregates equal to, or larger than the indicated sizes in response to LPS treatment of wild-type mice, neutrophil depleted mice, Kupffer cell depleted mice, and mice depleted of both neutrophils and Kupffer cells. *** *p*<0.001, ** *p*<0.01, * *p*<0.05.
(TIF)

Figure S6 **Circulating platelet-neutrophil aggregates.** Time lapse images of a circulating platelet-neutrophil aggregate. The circulating neutrophil is denoted by yellow arrows and the associated platelet aggregate is denoted by white arrows (note: the platelet aggregates appear ahead of the neutrophil due to the sequential capture of individual fluorescent channels). Platelets labelled with PE-conjugated anti-CD49b (red); neutrophils labelled with Alexa Fluor 647-conjugated anti-Gr-1 (blue); green is liver autofluorescence to illustrate vessels. Sequential images represent 2.3 s intervals. Scale bar, 20 µm.
(TIF)

Figure S7 **Flow cytometric analysis of blood from CD41-YFP^ki/+^ mice.** Representative histograms from four individual CD41-YFP**^ki/+^** mice demonstrating variability in the percentage of YFP+ platelets between animals. Histograms pre-gated on size and for positive PE-conjugated anti-CD41 staining.
(TIF)

Figure S8 **Area occupied by neutrophils and Kupffer cells in the livers untreated and LPS-treated mice.** The area corresponding to neutrophil staining (GR1+ staining) and Kupffer cell staining (F4/80+ staining) were measured in 12 representative fields of view (FOV) from untreated mice and mice treated with 1 mg/kg LPS intravenously 4 h earlier. *** *p*<0.001, n.s. not significant.
(TIF)

Movie S1 **In vivo labelling of peripheral platelets by intravenous injection of PE-conjugated anit-CD49b.** Intravital visualization of circulating platelets within the liver microvasculature of an untreated C57BL/6 mouse using spinning-disk confocal fluorescence microscopy. Platelets labelled with PE-conjugated anti-CD49b (red); neutrophils labelled with Alexa Fluor 647-conjugated anti-Gr-1 (blue). Objective: UPLANSAPO 10×/0.40. Scale bar, 40 µm.
(MOV)

Movie S2 **Platelet interactions in the liver of a control mouse.** Platelets labelled with PE-conjugated anti-CD49b can be seen circulating through the liver sinusoids of an untreated C57BL/6 mouse. These platelets are seen to form frequent, but brief interactions with the sinusoid walls and few adherent neutrophils. A small number of platelets appear to adhere to the liver sinusoids even under control conditions. Platelets labelled with PE-conjugated anti-CD49b (red); neutrophils labelled with Alexa Fluor 647-conjugated anti-Gr-1 (blue). Objective: UPLANSAPO 20×/0.70. Scale bar, 20 µm.
(MOV)

Movie S3 **Platelet behaviour in the microvasculature of the ear in a control mouse.** Although no interactions between platelets and neutrophils are seen in the ear microvasculature of an untreated C57BL/6 mouse, a small number of brief (touching-down) interactions between platelets and the endothelium can be observed. Platelets labelled with PE-conjugated anti-CD49b (red); neutrophils labelled with Alexa Fluor 647-conjugated anti-Gr-1 (blue); endothelium labelled with Alexa Fluor 488-conjugated anti-CD31 (green). Objective: XLUMPPlanFI 20×/0.95W. Scale bar, 20 µm.
(MOV)

Movie S4 **Absence of CD49b labelled platelets in a thromboyte depleted mouse.** Liver intravital microscopy in a mouse treated for 18 h with anti-thrombocyte serum (100 µl/mouse i.v.). Although a CD49b labelled NK cell is visible (white arrow), there is an absence of the small adherent and circulating CD49b+ particles confirming the identity of these objects as platelets. NK cell and platelets labelled with PE-conjugated anti-CD49b (red); neutrophils labelled with Alexa Fluor 647-conjugated anti-Gr-1 (blue). Objective: UPLANSAPO 20×/0.70. Scale bar, 20 µm.
(MOV)

Movie S5 **Platelet interactions and adhesion within the liver of an LPS treated mouse.** Intravital visualization of a liver from a C57BL/6 mouse 4 h after LPS treatment (1 mg/kg i.v.). Platelets can be seen interacting with the sinusoids and neutrophils that have been recruited to the liver, generating dynamic aggregates and sloughing off into circulation. Platelets labelled with PE-conjugated anti-CD49b (red); neutrophils labelled with Alexa Fluor 647-conjugated anti-Gr-1 (blue). Objective: UPLANSAPO 10×/0.40. Scale bar, 40 µm.
(MOV)

Movie S6 **4D imaging of platelet neutrophil interactions within the liver of an LPS treated mouse.** Sequential intravital z stacks were imaged within the liver of an LPS (4 h, 1 mg/kg i.v.) treated mouse, compiled and rendered as 3D opacity models using the Volocity software package. Platelets can be seen to be interacting with, and adhering to neutrophils within the liver sinusoids. Platelets were labelled with PE-conjugated anti-CD49b (rendered red); neutrophils were labelled with Alexa Fluor 647-conjugated anti-Gr-1 (rendered blue). Objective: UPLANSAPO 20×/0.70. Grid 25.7 µm.
(MOV)

Movie S7 **Dynamic platelet interactions with neutrophils within the liver of an LPS treated mouse.** Platelet interactions and adhesion within the liver of an LPS (4 h, 1 mg/kg i.v.) treated C57BL/6 mouse. Platelets can be seen interacting with, adhering to, and “flowing” across the surface of the neutrophils. These platelets are also observed to from unstable and dynamic aggregates on downstream edge of the cell before tearing off into the circulation (white arrow). Platelets labelled with PE-conjugated anti-CD49b (red); neutrophils labelled with Alexa Fluor 647-conjugated anti-Gr-1 (blue). Objective: UPLANSAPO 20×/0.70. Scale bar, 20 µm.
(MOV)

Movie S8 **Circulating platelet-neutrophil aggregates within the liver of an LPS treated mouse.** Intravital visualization of a liver from a C57BL/6 mouse 4 h after LPS treatment (1 mg/kg i.v.). Two neutrophils coated in adherent platelets can be seen circulating through the liver microvasculature (neutrophils enter FOV at t = 4 s and t = 11 s) (note: the platelet aggregates appear ahead of the neutrophil due to the sequential capture of individual fluorescent channels). Platelets labelled with PE-conjugated anti-CD49b (red); neutrophils labelled with Alexa Fluor 647-conjugated anti-Gr-1 (blue); green is liver autofluorescence to illustrate vessels. Objective: UPLANSAPO 20×/0.70. Scale bar, 20 µm.
(MOV)

Movie S9 **Platelet behaviour within the liver of an untreated CD41-YFP^ki/+^ mouse.** Intravital visualization of the liver of an untreated CD41-YFP**^ki/+^** mouse. Although some platelets are seen to interact with the liver sinusoids and neutrophils, few adherent platelets are observed. Neutrophils labelled with Alexa Fluor 647-conjugated anti-Gr-1 (blue); YFP+ platelets and liver autofluorescence (green). Objective: UPLANSAPO 20×/0.70. Scale bar, 20 µm.
(MOV)

Movie S10 **Visualization of platelet adhesion in an LPS treated CD41-YFP^ki/+^ mouse.** Intravital visualization of YFP+ platelets interacting with, and adhering to neutrophils within the liver of an LPS (4 h, 1 mg/kg i.v.) treated CD41-YFP**^ki/+^** mouse. Neutrophils labelled with Alexa Fluor 647-conjugated anti-Gr-1 (blue); YFP+ platelets and liver autofluorescence (green). Objective: UPLANSAPO 20×/0.70. Scale bar, 20 µm.
(MOV)

Movie S11 **Platelet interactions with Kupffer cells within the liver of an LPS treated mouse.** Visualization of YFP+ platelets interacting with, and adhering to sinusoids, Kupffer cells and neutrophils within the liver of an LPS (4 h, 1 mg/kg i.v.) treated CD41-YFP**^ki/+^** mouse. Kupffer cells labelled with PE-conjugated anti-F4/80 (red); neutrophils labelled with Alexa Fluor 647-conjugated anti-Gr-1 (blue); YFP+ platelets and liver autofluorescence (green). Objective: UPLANSAPO 20×/0.70. Scale bar, 20 µm.
(MOV)

Movie S12 **Platelet behaviour in arterioles and postcapillary venules of the brain in a control mouse.** Intravital visualization of an arteriole (bright CD31 staining) and postcapillary venule (dim CD31 staining) in the brain of an untreated C57BL/6 mouse. No adherent neutrophils and few platelet-endothelium interactions can be observed. Platelets labelled with PE-conjugated anti-CD49b (red); neutrophils labelled with Alexa Fluor 647-conjugated anti-Gr-1 (blue); endothelium labelled with Alexa Fluor 488-conjugated anti-CD31 (green). Objective: Objective: XLUMPPlanFI 20×/0.95W. Scale bar, 20 µm.
(MOV)

Movie S13 **Platelet behaviour in arterioles and postcapillary venules of the brain in an LPS treated mouse.** Intravital visualization of an arteriole (bright CD31 staining) and postcapillary venule (dim CD31 staining) in brain of an LPS (4 h, 1 mg/kg i.v.) treated C57BL/6 mouse. Numerous adherent neutrophils, platelet-neutrophil, and platelet-endothelial interactions can be seen within the postcapillary venule. Platelets labelled with PE-conjugated anti-CD49b (red); neutrophils labelled with Alexa Fluor 647-conjugated anti-Gr-1 (blue); endothelium labelled with Alexa Fluor 488-conjugated anti-CD31 (green). Objective: XLUMPPlanFI 20×/0.95W. Scale bar, 20 µm.
(MOV)

Movie S14 **Platelet behaviour in postcapillary venules of the cremaster muscle in a control mouse.** Postcapillary venule in a C57BL/6 mouse imaged under control conditions 4 h after local injection of the cremaster with saline. Although no interactions between platelets and rolling, or circulating neutrophils are seen, some short term (touching-down) interactions of the platelets with the endothelium can be observed. Platelets labelled with PE-conjugated anti-CD49b (red); neutrophils labelled with Alexa Fluor 647-conjugated anti-Gr-1 (blue); endothelium labelled with Alexa Fluor 488-conjugated anti-CD31 (green). Objective: UPLANFL N 10×/0.30W. Scale bar, 40 µm.
(MOV)

Movie S15 **Platelet-neutrophil interactions in postcapillary venules of the cremaster muscle after local LPS stimulation.** Postcapillary venule in a C57BL/6 mice imaged 4 h after local injection of the cremaster with LPS (0.5 µg/kg) in saline. Platelets are seen to be adhering to, and interacting with endothelium and both adherent and circulating neutrophils (white arrow – appears at t = 18 s). Platelets labelled with PE-conjugated anti-CD49b (red); neutrophils labelled with Alexa Fluor 647-conjugated anti-Gr-1 (blue); endothelium labelled with Alexa Fluor 488-conjugated anti-CD31 (green). Objective: XLUMPPlanFI 20×/0.95W. Scale bar, 20 µm.
(MOV)
