# Supplementary figures and images for: The Use of Spinning-Disk Confocal Microscopy for the Intravital Analysis of Platelet Dynamics in Response to Systemic and Local Inflammation
Source: PLoS One. 2011 Sep 19;6(9):e25109. doi: 10.1371/journal.pone.0025109 (PMC3176312; doi:10.1371/journal.pone.0025109)

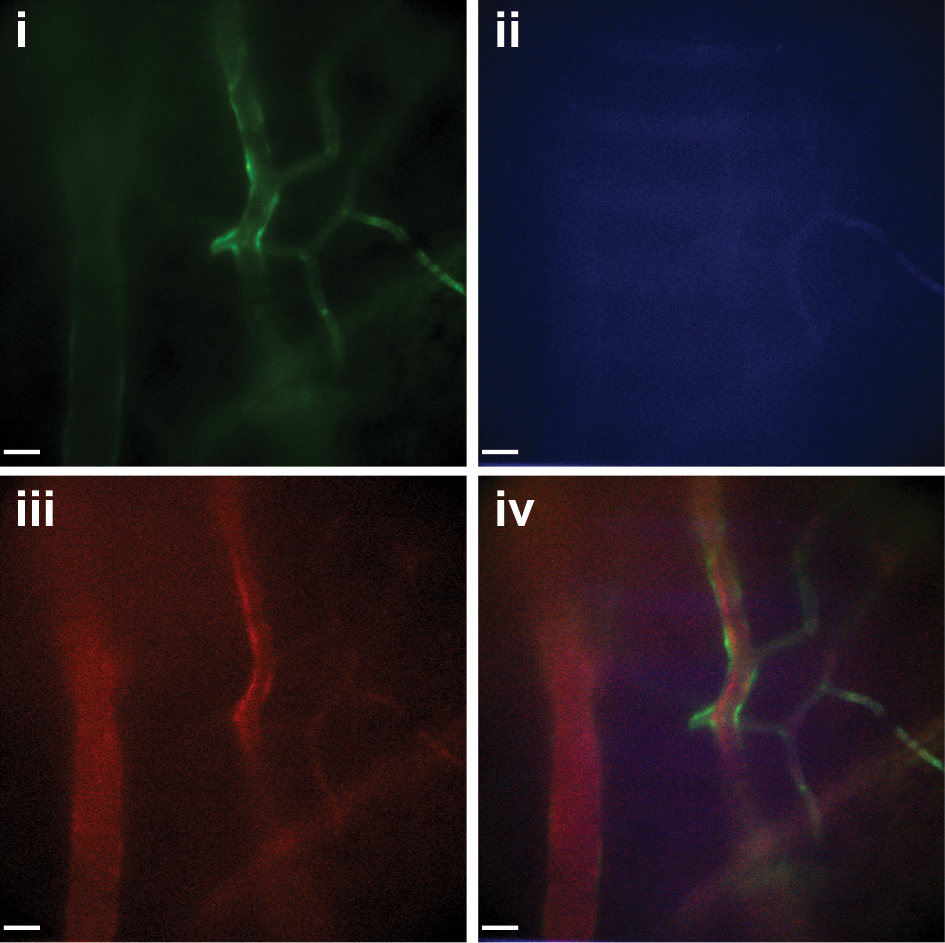

Supplement: Figure S1 — Intravital visualization of platelets labelled with PE-conjugated anti-CD49b within the mouse ear. Spinning-disk confocal-microscopic imaging of mouse ear microvasculature in untreated mice. Visualization of endothelium with Alexa Fluor 488-conjugated anti-CD31 (i), neutrophils labelled with Alexa Fluor 647-conjugated anti-Gr-1 (ii), platelets stained with PE-conjugated anti-CD49b (iii), and multi-channel overlay (iv). No adherent and few endothelial-interacting platelets were seen in the mouse ear, a tissue visualized without surgical intervention, under control conditions. All scale bars, 20 µm. (TIF) [file pone.0025109.s001.tif]

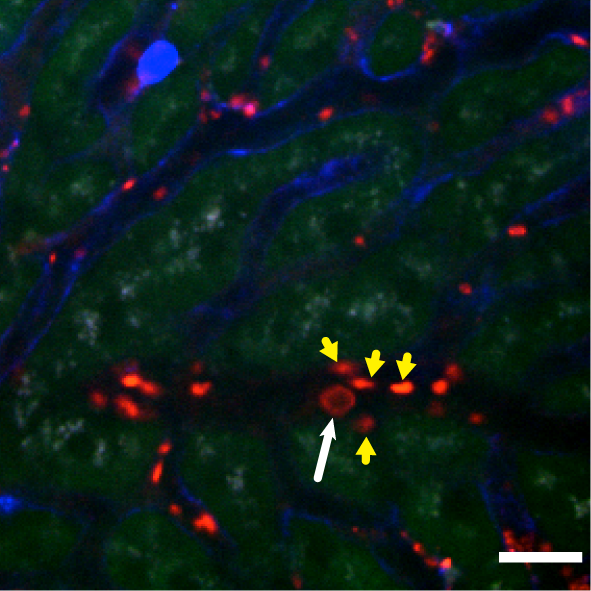

Supplement: Figure S2 — In vivo labelling of mouse NK cells by intravenous injection of the anti-CD49b antibody HMα2. Intravenous administered PE-conjugated anti-CD49b (red) also labels NK cells in vivo (white arrow). Their larger size and less intense staining makes labelled NK cells easily discernable from nearby platelets (small yellow arrows). Neutrophils are labelled with Alexa Fluor 647-conjugated anti-Gr-1 (blue); green is liver autofluorescence to illustrate vessels. Scale bar, 20 µm. (TIF) [file pone.0025109.s002.tif]

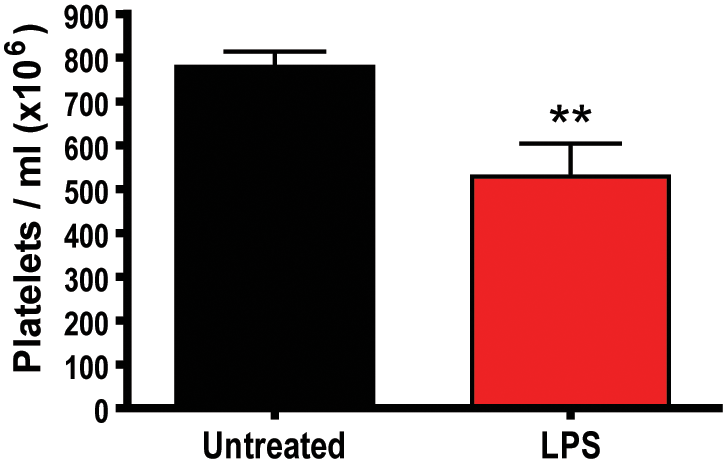

Supplement: Figure S3 — Intravenous treatment with LPS results in a reduction in circulating platelets. Circulating platelet counts from untreated and mice treated with 1 mg/kg LPS intravenously for 4 h. ** p = 0.01. (TIF) [file pone.0025109.s003.tif]

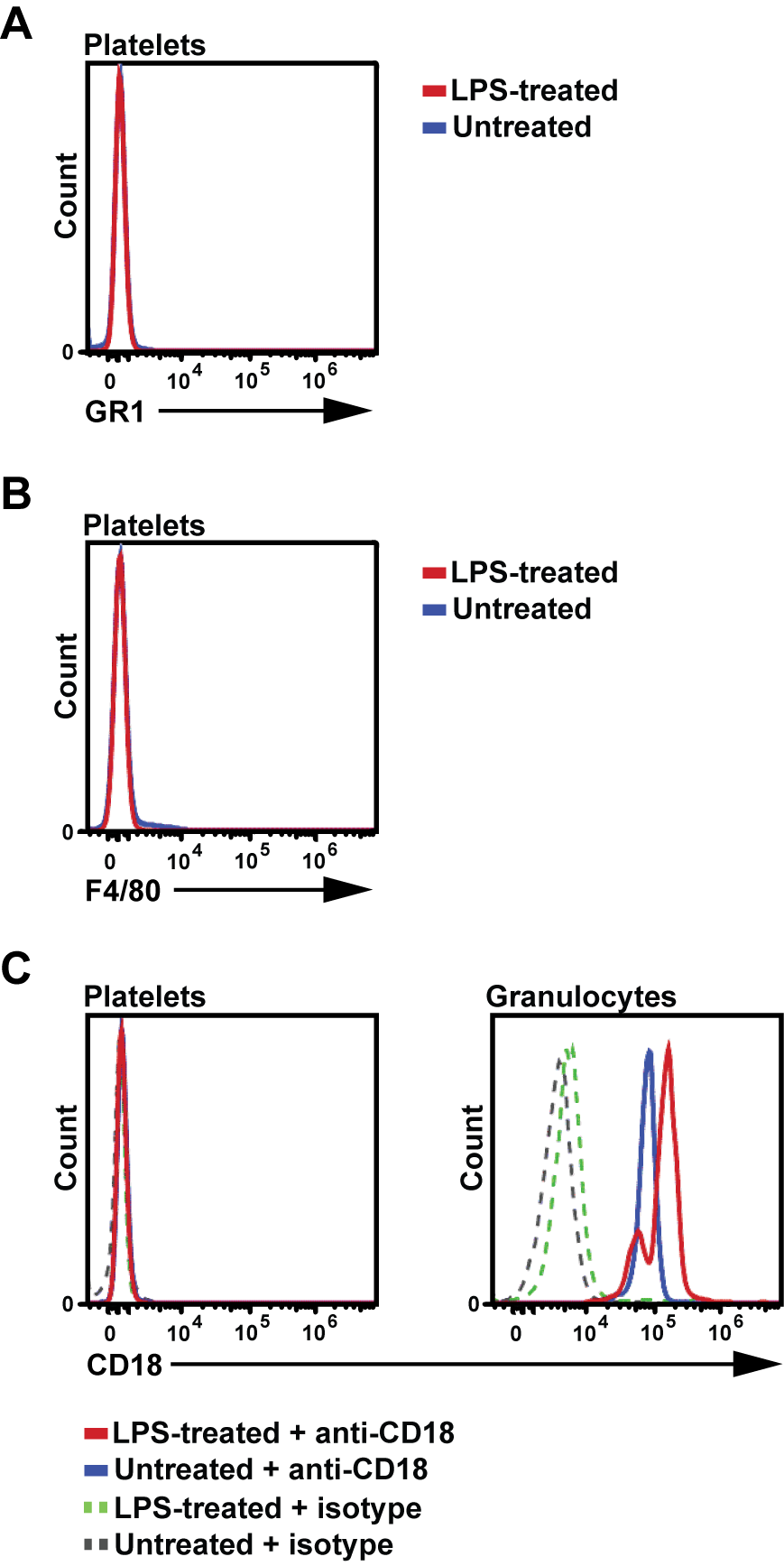

Supplement: Figure S4 — Flow cytometric analysis of platelets from untreated and LPS-treated mice. Absence of neutrophil associated (GR1) (A) or macrophage associated (F4/80) (B) surface markers on platelets from untreated or LPS-treated mice. Staining for CD18 expression on platelets (C, left panel) and granulocytes (D, right panel) obtained from untreated or LPS-treated mice. (TIF) [file pone.0025109.s004.tif]

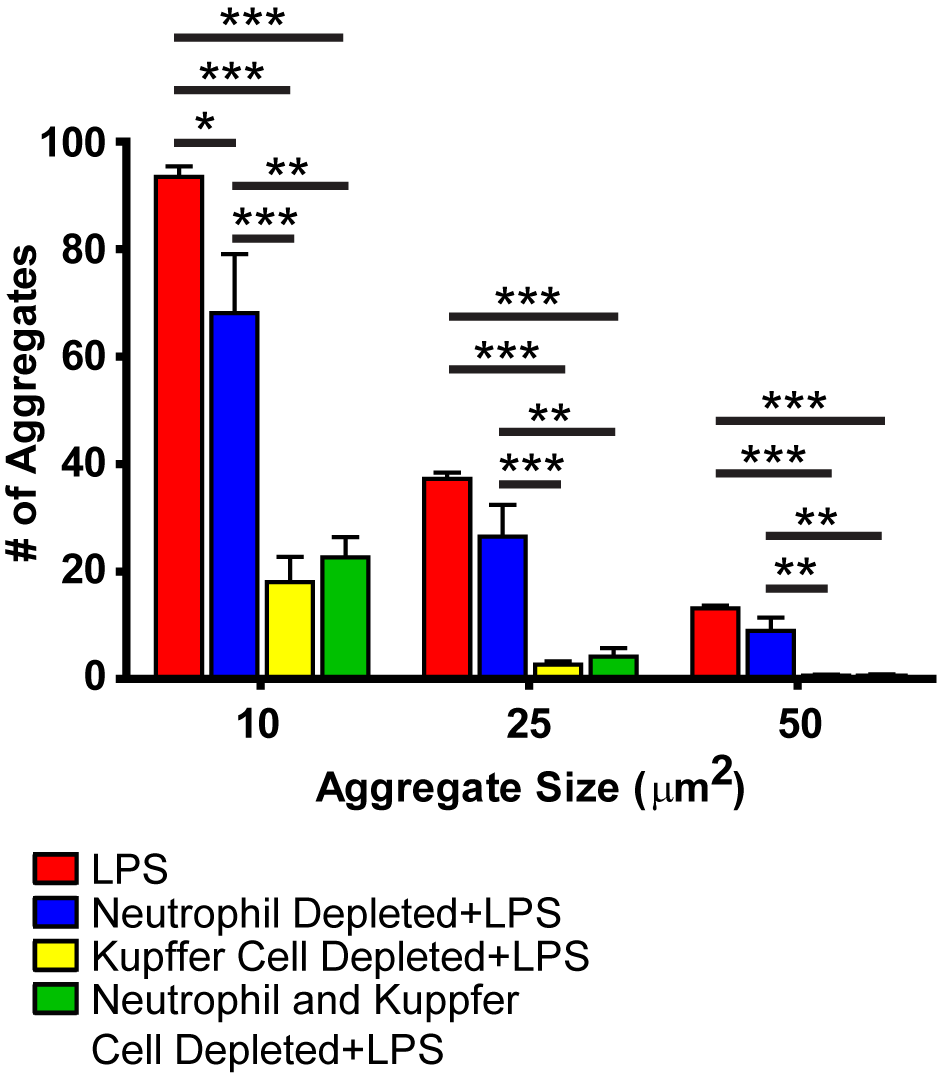

Supplement: Figure S5 — Effect of neutrophil and Kupffer cell depletion on platelet aggregation in the mouse liver in response to LPS treatment. Quantification of the number of platelet aggregates equal to, or larger than the indicated sizes in response to LPS treatment of wild-type mice, neutrophil depleted mice, Kupffer cell depleted mice, and mice depleted of both neutrophils and Kupffer cells. *** p<0.001, ** p<0.01, * p<0.05. (TIF) [file pone.0025109.s005.tif]

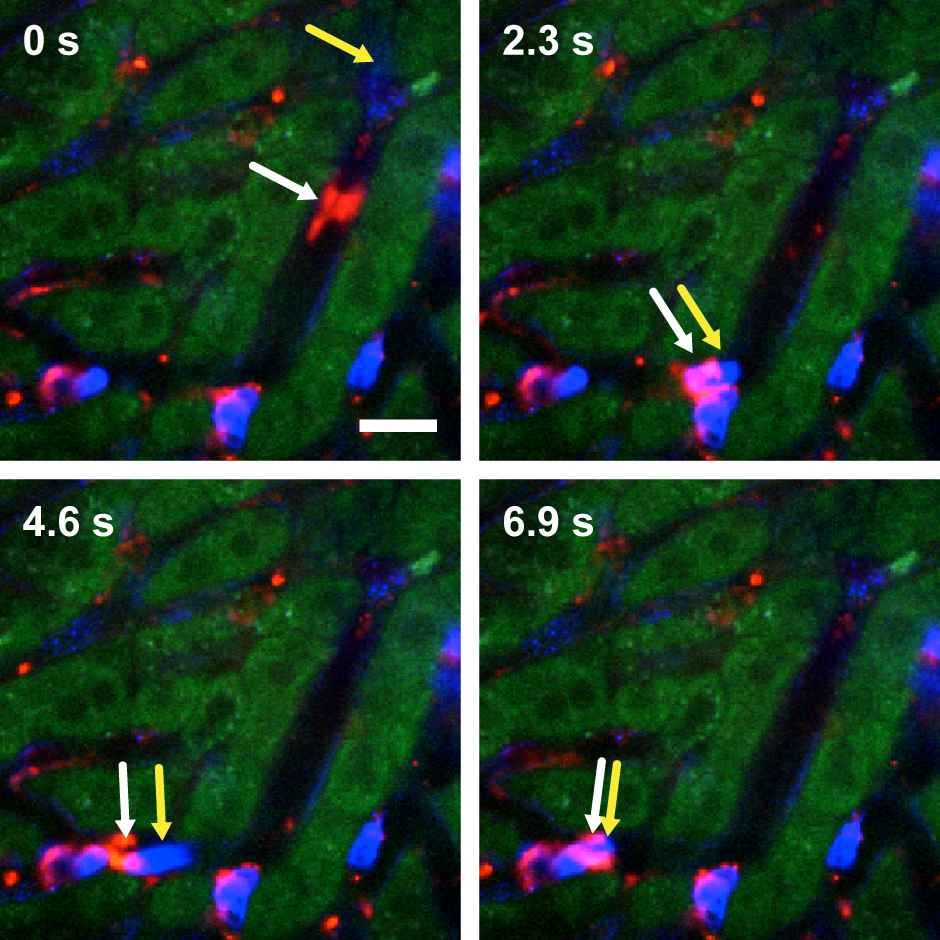

Supplement: Figure S6 — Circulating platelet-neutrophil aggregates. Time lapse images of a circulating platelet-neutrophil aggregate. The circulating neutrophil is denoted by yellow arrows and the associated platelet aggregate is denoted by white arrows (note: the platelet aggregates appear ahead of the neutrophil due to the sequential capture of individual fluorescent channels). Platelets labelled with PE-conjugated anti-CD49b (red); neutrophils labelled with Alexa Fluor 647-conjugated anti-Gr-1 (blue); green is liver autofluorescence to illustrate vessels. Sequential images represent 2.3 s intervals. Scale bar, 20 µm. (TIF) [file pone.0025109.s006.tif]

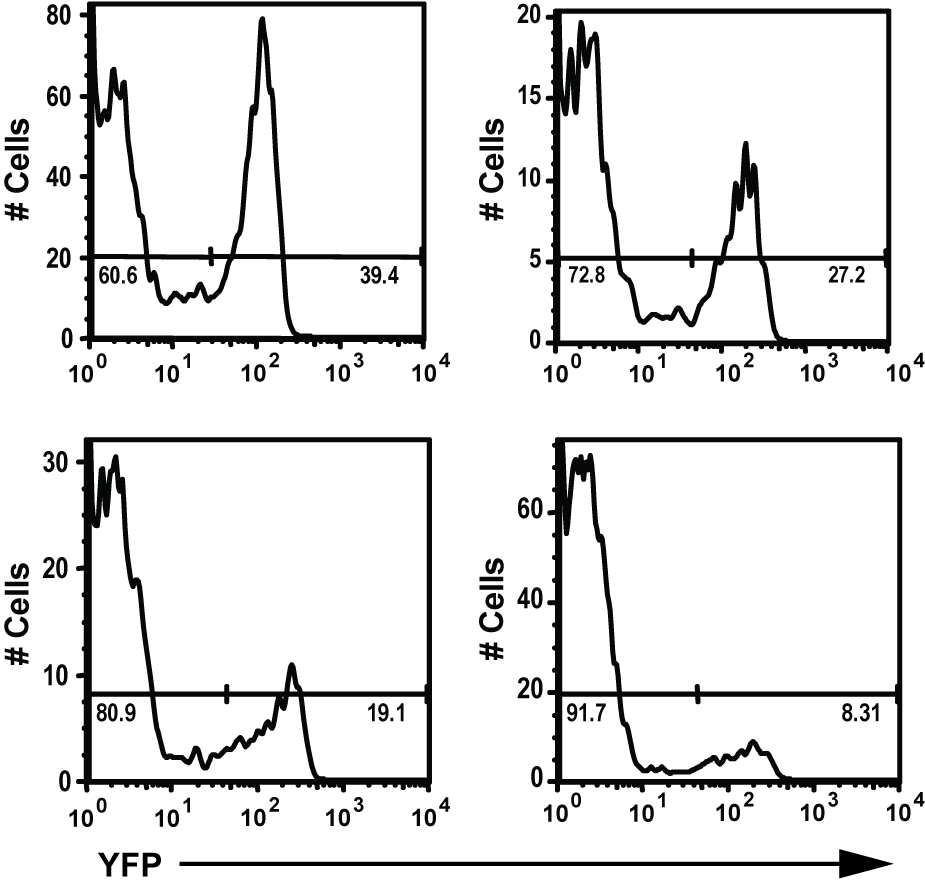

Supplement: Figure S7 — Flow cytometric analysis of blood from CD41-YFPki/+ mice. Representative histograms from four individual CD41-YFPki/+ mice demonstrating variability in the percentage of YFP+ platelets between animals. Histograms pre-gated on size and for positive PE-conjugated anti-CD41 staining. (TIF) [file pone.0025109.s007.tif]

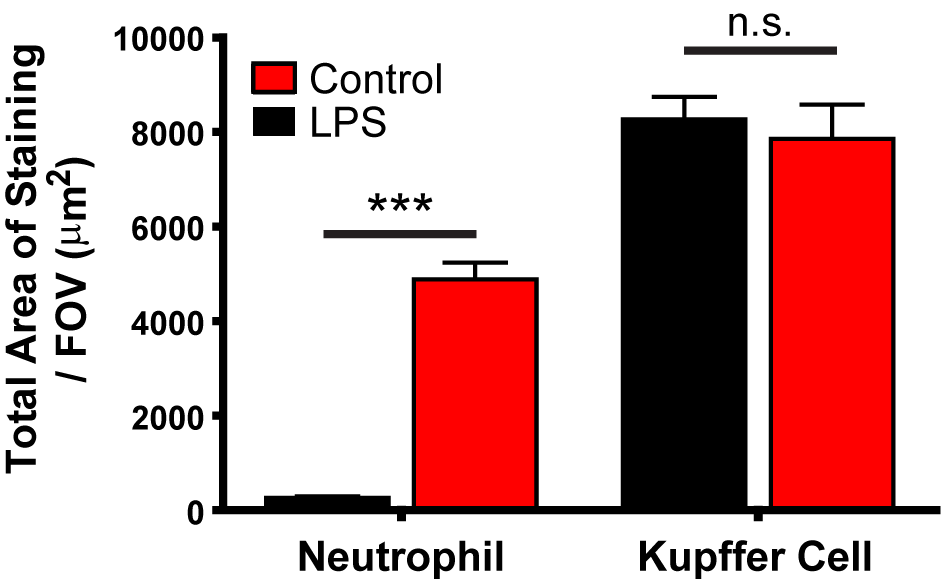

Supplement: Figure S8 — Area occupied by neutrophils and Kupffer cells in the livers untreated and LPS-treated mice. The area corresponding to neutrophil staining (GR1+ staining) and Kupffer cell staining (F4/80+ staining) were measured in 12 representative fields of view (FOV) from untreated mice and mice treated with 1 mg/kg LPS intravenously 4 h earlier. *** p<0.001, n.s. not significant. (TIF) [file pone.0025109.s008.tif]
